# Recent Progress in the Design and Medical Application of In Situ Self-Assembled Polypeptide Materials

**DOI:** 10.3390/pharmaceutics13050753

**Published:** 2021-05-19

**Authors:** Tian-Tian Wang, Yi-Yi Xia, Jian-Qing Gao, Dong-Hang Xu, Min Han

**Affiliations:** 1 Department of Pharmacy, The Second Affiliated Hospital, School of Medicine, Zhejiang University, Hangzhou 310058, China; 21719002@zju.edu.cn; 2 Institution of Pharmaceutics, College of Pharmaceutical Sciences, Zhejiang University, Hangzhou 310058, China; 22060367@zju.edu.cn (Y.-Y.X.); gaojianqing@zju.edu.cn (J.-Q.G.)

**Keywords:** polypeptide, self-assembly, medical applications

## Abstract

Inspired by molecular self-assembly, which is ubiquitous in natural environments and biological systems, self-assembled peptides have become a research hotspot in the biomedical field due to their inherent biocompatibility and biodegradability, properties that are afforded by the amide linkages forming the peptide backbone. This review summarizes the biological advantages, principles, and design strategies of self-assembled polypeptide systems. We then focus on the latest advances in in situ self-assembly of polypeptides in medical applications, such as oncotherapy, materials science, regenerative medicine, and drug delivery, and then briefly discuss their potential challenges in clinical treatment.

## 1. Introduction

Polypeptides are short chains composed of a number of amino acid monomers arranged in a specific order and bound by peptide bonds. They are widely found in organisms and have important biological functions. Compared to proteins with large molecular weight and complex structure, polypeptides have unique advantages, such as simple structure and easy synthesis, good biocompatibility, being non-toxic and self-biodegradable, easy to function and modify, etc., and are ideal biomedical materials. The past five years have seen great strides in the synthesis of polypeptides with a number of approaches reported for obtaining controlled polypeptides from varied initiators in shorter timeframes, such as classical ring opening polymerization of α-amino acid, which plays an important role in the wide range of application [1,2,3]. However, unsatisfactory bioavailability and instability limit its further application in clinical medicine. Molecular self-assembly is a spontaneous process guided by various non-covalent interactions, including electrostatic interactions, hydrophobic effects, aromatic stacking, and hydrogen bonding [4,5,6]. The self-assembly of peptide molecules into proteins with different functions is crucial to the life activities of cells. For example, the formation of actin filaments and microtubules will affect the movement and migration of cells [7,8], and the formation of apoptotic bodies [9,10] is closely related to programmed cell death; as well, inflammatory bodies [11] will also affect the body’s immune response to a certain extent. Similarly, the wrong folding of polypeptides into proteins will also cause body disease, such as neurodegenerative diseases resulting from the fibrous assemblies of abnormal proteins [12]. These phenomena have aroused great attention to the process of protein assembly, which not only clarified the cell function and disease mechanism to a certain extent but also promoted the application of the biological functions of artificial nanostructured materials in biology and medicine.

Inspired by these widespread principles in nature [13] (e.g., myoglobin, ferritin, nucleosome, collagen, double-strand DNA), scientists devoted themselves to utilize various biocompatible materials and more convenient synthetic methods, including aqueous ring-opening polymerization-induced self-assembly [14], to create rational self-assembled systems with tunable properties and targeted application [15,16]. In 1993, Zhang et al. designed and synthesized an ion-complementary polypeptide EAKl6 consisting of 16 amino acid residues that can self-assemble in an aqueous solution to form a stable hydrogel membrane visible to the naked eye [17]. In the same year, Ghadiri designed a ring polypeptide consisting of eight amino acid residues. The polypeptide is self-assembled from β-folded structure and further highly integrated to form a hollow polypeptide nanotube [18]. Meanwhile, with improved understanding of sequence-to-structure relationships, Woolfson and others have made great efforts to design or explore more innovative assemblies so as to generate new protein functions based on the α-helical coiled coils, which are ubiquitous protein–protein-interaction domains. They charted a potential new territory in coiled-coil assemblies and α-helical barrels [19,20]. Since then, the design and application of self-assembled polypeptides has become a research hotspot in biomedicine, material chemistry, and other scientific fields [21,22,23,24,25].

**Scheme 1:**
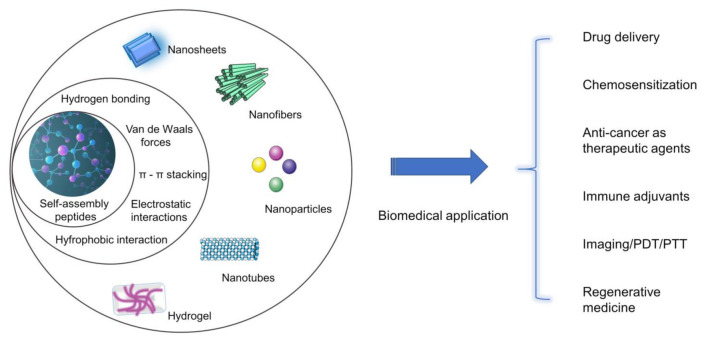
Schematic illustration of development of in vivo self-assembled polypeptides, including assembly driving forces, morphologies, bioeffects, and biomedical applications.

In this review, we summarized the classification of self-assembled polypeptides and the main applications in oncotherapy, materials science, regenerative medicine, and drug delivery. At the same time, we accordingly highlighted major advantages and current design strategies of peptide nanostructures with a discussion of upcoming goals and therapeutic potential in the future of these versatile materials (Scheme 1).

## 2. Classification and Assembly Principle of Self-Assembled Polypeptides

Molecular self-assembly is a spontaneous process guided by non-covalent interactions and occurs widely in biological systems, such as the formation of DNA double helical structures. In the same way, peptides can form specific peptide structures under certain conditions or trigger spontaneous self-assembly via hydrogen bonding and electrostatic hydrophobic interaction. The copolymer architectures show a low molecular aggregation with excellent performance, which makes the peptide self-assembly become immeasurable potential biomaterials in many fields. Polypeptide self-assembly can occur by spontaneous or triggered modes [26]. Under suitable conditions, polypeptides can spontaneously form assemblies in aqueous solutions or self-assemble into novel and functional materials triggered by changes in the external environment, such as pH, temperature, ion concentration, etc. Because of the reversibility and specificity of the process, current research in nanotechnology focuses on the construction of trigger-type polypeptide self-assembly systems [27].

Generally, these stimuli can be divided into two modes: internal stimulation and external stimulation. External stimulation, including electromagnetic, light, radiation, ultra-sound, etc., can guide active molecular peptides to self-assemble in the diseased area of the body [28]; internal stimulation is based on the intrinsic difference between the physiological part and the diseased tissue to trigger the self-assembly of the polypeptide, such as low pH between tissues, high intracellular GSH concentration or certain enzymes (such as matrix metalloproteinases), and the hypoxic environment of tumor tissues. At the cellular level, pH sensitivity can trigger the release of transported drugs into late endosomes or lysosomes or promote the escape of nanocarriers from lysosomes into the cytoplasm. Furthermore, at the tissue level, designers can use pathologies or specific microenvironmental changes related to tumor disease characteristics (such as ischemia, inflammatory diseases, or infections) to maximize the therapeutic advantages of responsive polypeptide carriers [29]. In addition, changes in some systemic biochemical parameters could also be used to stimulate the design of response nanocarriers, such as pH gradients, in and out of cells and in the gastrointestinal tract. Stimulus-responsive nanocarriers can control and adjust the position and time of polypeptide self-assembly, thereby prompting nanoformulations to accurately deliver and release drugs at desired targets with fewer adverse reactions and ultimately realizing a self-controlled drug release mode.

### 2.1. Spontaneous Self-Assembled Polypeptides

The traditional amphiphilic concept is introduced into the spontaneous self-assembled polypeptide system to obtain polypeptide derivatives similar to surfactants or liposomes, which are called amphiphilic polypeptides (peptide amphiphile). When dissolved in aqueous solutions, the exposed hydrophilic segment interfaces with the water molecule, while the hydrophobic segment concentrates inward, and they repeatedly form complementary ionic bonds on hydrophilic surfaces. After that, they transform into various secondary structures (including -helical -folding -hairpins) so as to form copolymers spontaneously. Furthermore, the micellar vesicle, monolayer membrane, nanofiber, nanotube, and other assemblies can be obtained by adjusting the structure and length. For example, the CCL2 binding region from a G protein coupled receptor was introduced to the self-assembled domain(K–SLSLSLSLSLSL–K). In the aqueous medium, the self-assembling peptide [SLaM: K–(SL)_6_–K–G–WKNFQTI] spontaneously forms β-sheet based supramolecular nanofibers step by step, which were stabilized by a hydrophobic core formed by leucine side chains and hydrogen bonds along the principal axis [30]. Additionally, Jin et al. also reported an amphiphilic polypeptide KFAK (H_2_N–KKFAFAFAKK–COOH) that could form nanofibers by hydrogen bonds between peptide skeletons and the hydrophobic interaction between side-chain groups. Then, small molecule 4,4-bipyridine (4Bpy) was introduced for adjustment to assemble a two-dimensional nanosheet structure from a one-dimensional fibril [31].

### 2.2. Trigger-Type Self-Assembled Polypeptides

Changes in the external environment drive self-assembly of peptides and endow them with unique functions. These triggered self-assembly peptides are widely used in tissue engineering, drug delivery systems, intelligent biomaterials, and other fields. Factors that regulate the occurrence of self-assembly processes include pH, temperature, photo, enzymes, metal ions, etc. The regulation of pH value in the physiological environment to the polypeptide self-assembly system is the result of protonation and deprotonation of basic and acidic amino acids [27]. Martin et al. studied a dual-regulated self-assembled cyclic antimicrobial peptide nanotube that was sensitive to both the presence of a lipid membrane and the pH of the aqueous media. Under these conditions of low pH value and a lipid bilayer, the antibacterial peptide can be self-assembled into nanotubes with cavity structures, which can allow the permeation of water and prevent that of ions. Based on the effect of ionization on self-assembly behavior, higher pH values were expected to cause the change of channel characteristics, including the protonation leading to the disintegration of nanostructures and ion to double hydrophobic nuclear translocation, so as to realize the function of the self-assembled material to kill target cells [32].

Similarly, the polypeptide self-assembly process is also sensitive to temperature changes. Temperature increase often leads to the expansion of the secondary structure of the polypeptide, which, finally, interrupts its function. Therefore, thermo-responsive peptides may be designed as biomaterials that are regulated by environmental temperature changes for drug release or regenerative medicine, in which thermosensitive sol-gel systems are of concern. In the sol phase, the therapeutic drugs can be mixed with sol polypeptides by simple and environmentally friendly methods. After injection, temperature change causes in situ self-assembly of solution into hydrogel, which is not only simple and safe but also can be used as a sustained release repository for local drug delivery [33]. For example, Cao et al. developed a thermo-sensitive reversible sol-gel polypeptide hydrogel system composed of poly-isopropylacrylamide and antimicrobial peptides. The system prepared injectable hydrogels that induced molecular conformation changes by heat under physiological conditions and triggered in-situ sol-gel transformation. Meanwhile, G(IIKK)_3_I–NH_2_ can be released from the hydrogel in a continuous linear fashion, allowing for minimally invasive drug delivery.

Enzymes can effectively catalyze many biochemical reactions under physiological conditions. In addition, the level of enzyme expression is closely related to disease. Since certain enzymes are often overexpressed in abnormal sites of disease, such as cancer cells, EISA (enzymatic self-assembly of peptides) may occur at the site of disease or in abnormal cells. EISA in situ supramolecular nanomaterials are a safe and efficient preparation method for diagnosis and treatment of diseases. Phosphorylation modified an antitumor peptide YSV with the effect of histone deacetylase inhibitor into a prodrug (NapGDFDFpYSV), and it can be transformed into EISA hydrogel at the site of alkaline phosphorylase overexpression, which effectively improves the anticancer efficiency [34]. Apart from these, other stimulus-responsive strategies have also been extensively developed in recent years.

Although the self-assembly of small molecule peptides is diverse, the process of self-assembly has the following common points: (1) the self-assembled building block peptides only form nanometers in specific parts, such as target tissues, target organs, or target cells; (2) specific environmental conditions trigger the self-assembly process, such as the endogenous physiological characteristics of the cell, including a high concentration of enzymes inside the cell or low pH inside the lysosome and the microenvironment of the target tissue, including hypoxia in the tumor tissue or other stimulus responsiveness conditions; (3) the formation of nanostructures can be observed at the target site and lead to changes in cell structure or function to facilitate identification to a certain extent. With the in-depth understanding of the physiological differences between healthy tissues and diseased tissues and the establishment of in vitro stimulus responsiveness evaluation standards, researchers may discover more unknown and powerful internal stimuli and apply them in the design of nanocarriers in the future. Until then, responsive peptide nanocarriers have become more potential therapeutic biomaterials (Table 1).

## 3. Applications in Biomedicine

### 3.1. Drug Delivery System

Currently, chemotherapy, while still considered an effective therapy for cancers, has been limited by the severe side effects in clinical practices, such as its poor solubility, systemic instability, and lack of cancer specificity. In order to address the dilemma, scientists have made much effort to provide some strategies, specially nanoformulations, over the past few decades. As the emergence of new technologies, such as computer modeling, the development of drugs, biotechnology, and other bioactive agents, has been greatly accelerated, their specificity and activity of new dosage forms have also been improved successfully. The nano-drug delivery system can provide sufficient drug concentration to the therapeutic target while reducing the toxic side effects of drugs. However, current studies have found that nano-drug delivery systems, such as liposomes or inorganic nanocarriers, have certain clinical application risks [35,36,37]. For example, positively charged lipid nanocarriers can trigger a strong immune response. In addition, liposomes have technical limitations, as follows: poor reproducibility, stability, and low drug retention efficiency, as well as poor drug leaching control. However, drug delivery systems usually burst and leak the payloads in the initial stage and suffer from slow diffusion of the payloads in the treatment stage [38]. Polymer nanosystems may be used to alleviate the problems caused by the above formulations to a certain extent, but they are usually complicated in terms of surface functionalization to improve drug targeting and ineffective in most conditions. In addition, most nanostructures based on naturally-occurring polymers have the common problem of triggering unnecessary immune responses and are generally variable for different batches, which makes it difficult to predict their behavior in biological systems. Therefore, drug-controlled release and effective accumulation in tumor sites have been an obstacle for nano-drug delivery systems [39]. Polypeptide aggregates are good candidates for delivery vehicles due to the following characteristics: they self-assemble into core–shell aggregates spontaneously; water-soluble ionic polypeptides could be used to bind drugs bearing opposite charge [40]; and they contain a large amount of reactive groups that could be applied to conjugate drugs with labile chemical bonds [38].

**Figure 1:**
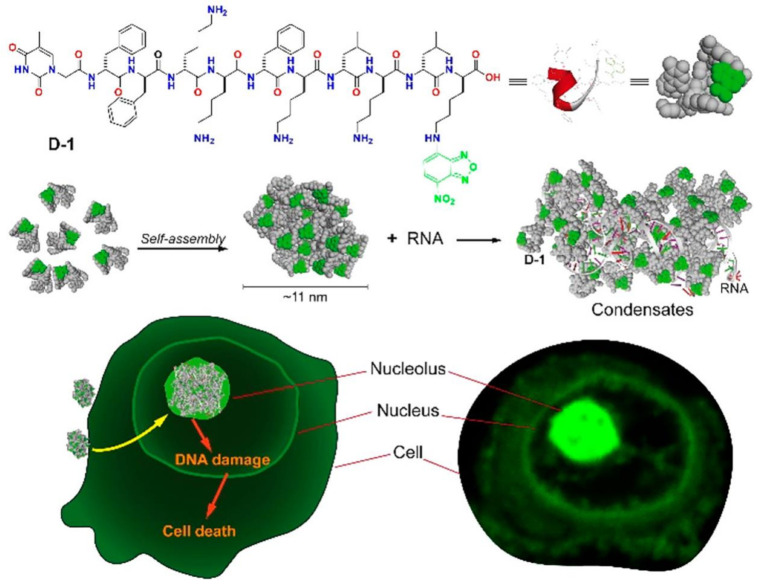
MolecularsStructures of D-1 and illustration of membraneless condensate formation of D-1 and RNA. Reproduced with permission from [43], American Chemical Society, 2019.

Though not being defined at the genetic level, nanoscale assemblies of the peptide are able to perform many critical biological functions, and previous studies have also high-lighted the significance in drug delivery [22,23]. It is widely known that self-assembly behavior of peptides occurred via non-covalent interactions, mainly, π-effects, van der Waal forces, and hydrogen bonding, which is extremely similar to the nucleobase stacking structure [23]. There is an excellent hypothesis that suggests the self-assembled peptides could deliver a DNA-intercalating chemotherapeutic, such as doxorubicin (Dox), locally to a solid tumor in a sustained manner based on the aforementioned principle. The Ade-FFF nucleo-peptide hydrogels have proved the above assumption. The self-assembling peptide system was perfectly capable of loading a high concentration of Dox up to 1 mM and demonstrated more linear and sustained Dox release profile compared with the previously reported delivery system, ultimately reducing tumor growth [41]. Furthermore, inspired by the long retention of large-scale structures in lesions, the doxorubicin–peptide conjugate nanoparticles (FDPC-NPs) were designed based on a morphological transformation strategy to achieve spatial accuracy and temporal persistence of drug delivery (Figure 1). The FDPC-NPs could maintain an appropriate size stable in blood circulation until entering the tumor stroma by the EPR effect and then responsively self-assemble into DPC-NFs in a mildly acidic microenvironment so as to stay in the tumor region for a long time and exert a sustained anti-tumor effect. This study will be a successful construction of a transformable drug delivery system (DDS) [42]. Meanwhile, as a benefit from the self-assembly mechanism, the D-peptide derivative enriched with lysine and hydrophobic residues self-assembled to form nanoparticles, which could interact with RNA to form membraneless condensates in the nucleolus and achieve nuclear targeting. The concept will illustrate a promising strategy based on the self-assembled D-peptides for targeting subcellular organelles [43].

**Figure 2:**
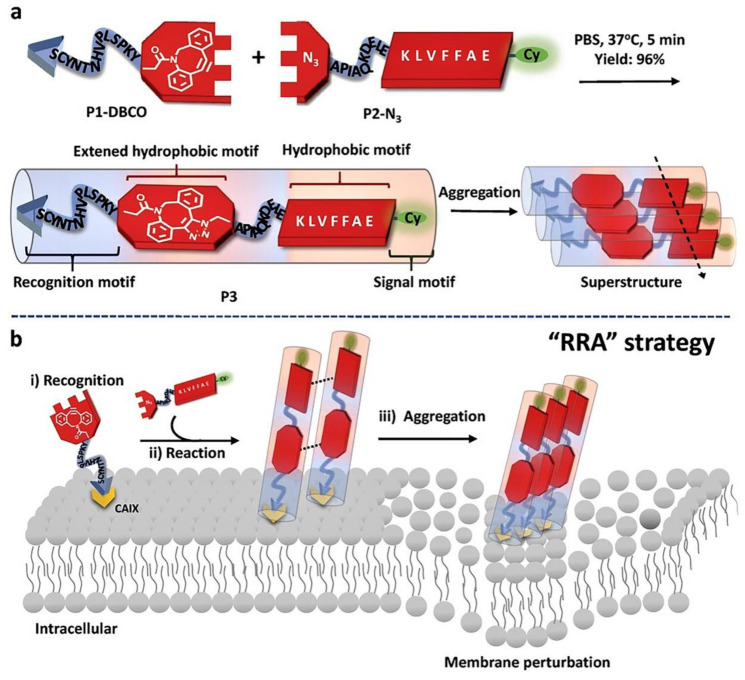
Schematic illustration of the molecular structure and the RRA cascade process. (**a**) The modular structure of DBCO-linked CAIX-targeting peptide (P1-DBCO) and azido-linked peptide labeled with cyanine dye (P2-N_3_). P1-DBCO and P2-N_3_ were monomeric states with hydrophilic–hydrophobic balance in aqueous solution, respectively. P2-N_3_ could effectively react with P1-DBCO to form a monomer peptide P3 at 37 °C for 5 min in PBS. The newly formed nitrogen heterocyclic structure of molecule P3 extended the hydrophobic unit, which broke the hydrophilic–hydrophobic balance, then accelerated aggregation to form the superstructure. (**b**) On the cellular level, P1-DBCO could specifically recognize renal cancer cells by targeting CAIX. Then, P2-N_3_ was added to react with P1-DBCO to form monomer peptide P3 on the cell membrane. Meanwhile, monomer P3 will aggregate to the superstructure that achieved membrane perturbation. Reproduced with permission from [45], John Wiley and Sons, 2019.

Multiple-drug-resistance (MDR) is still one main obstacle to systemic chemotherapy of tumors [44]. Recent studies have demonstrated that increasing the influx of chemodrugs is an effective plan for chemosensitization. For instance, Wang et al. developed the recognition–reaction–aggregation (RRA) cascaded strategy, a cascade process where P1-dibenzocyclooctyne (DBCO) performed specific recognition with cancer cells by the target head, and then P2-N_3_ was consequently introduced and reacted with P1-DBCO to form an aggregable self-assembled nanofiber P3 (Figure 2). In the final step, the nanofibers were able to specifically perturb the permeability of cell membranes and enhance chemo-drug sensitivity in vitro and in vivo [45]. Additionally, peptide–drug conjugate self-assembling nano-vesicles in situ and co-loading some immunostimulants might be a new avenue for tumor sensitization [46,47]. A series of founding experiments proved the breathtaking potentialities of self-assembled peptides in drug delivery and cancer therapies. Considering the characteristics of rapid clearance and low compliance of intravenous administration, changing the route may be another breakthrough in drug delivery. Heise et al. prepared the amphiphilic star polypept(o)ides base on star polypeptides with poly(lysine) arms, which showed excellent epithelial permeating capacity and disclosed a gene delivery platform [48]. At the same time, Lecommandoux and Deming designed a series of simple polysaccharide–block–polypeptide copolymers that could self-assemble into diverse aggregates and displayed a high affinity for cell mimicking of virus morphology. The functionalization of self-assembling peptides makes transporting therapeutic cargoes across the tissue barrier to target sites effectively possible [49,50,51,52,53,54,55].

Cardiovascular disease (including myocardial infarction) is the number one cause of death. Current treatments mostly use palliative therapy, which can delay the progression of heart failure but cannot regenerate healthy tissues. It was found that self-assembled peptides could be used to adapt to and simulate the natural microenvironment by adjusting its mechanical and biological activity characteristics. Many experimenters, inspired by the above results, have designed an experiment where a variety of growth factors and signal transduction molecules were embedded in self-assembled peptide hydrogels so as to strengthen behavior and organizational function of cells. For example, Singelyn and others have developed a myocardial-specific hydrogel that assembles itself in vivo. When injected in rats with ischemia-reperfusion model, the substance would increase the endogenous cardiomyocytes in the infarcted area and maintain heart function without causing arrhythmia at the time. Second, Hsieh et al. constructed injectable self-assembling peptide nanofibers combined with PDGF-BB in vitro to continuously deliver PDGF-BB to the myocardium at the injection site for 14 days. Research results showed that the nanofibers combined with PDGF-BB could reduce myocardial cell death and preserve the contractile function after myocardial infarction, as well as reduce the infarct area after ischemia/reperfusion [56]. These data indicate that the injectable nanofibers can accurately and continuously deliver drugs to the myocardium, thereby, exerting potential therapeutic benefits. The environmentally responsive self-assembled peptides nano-drug delivery system provides opportunities for minimally invasive treatment of clinical diseases.

### 3.2. Anti-Cancer as Therapeutic Agents

In the past decade, small-molecule nano-assemblies (or aggregates) have been widely used in high-throughput drug screening [57] and neurodegenerative diseases. In the process of studying the mechanism of neurodegenerative diseases, researchers found that early aggregates of misfolded non-disease-related proteins [58] and oligomers of disease-related proteins (such as Aβs) [59] showed similar cytotoxicity. In addition, studies have found that small-molecule self-assembled nanostructures can act as functional molecular entities in the cellular environment, such as chelating activating enzymes [60], inhibiting cell growth [23], and recruiting and retaining mRNA to form cell-free RNA particles [61]. These results not only show the progress of the important mechanism of aggregate cytotoxicity but also suggest that people can use these peptide or protein aggregates to induce apoptosis of cancer cells so as to slow down or eliminate the occurrence and development of tumors. However, the anti-tumor mechanism of nanofibers is still unclear. We found that the fibrous structures formed by the self-assembly of polypeptides aggregate in target tissues or target cells may interact with various intracellular proteins, such as actin and vimentin, thereby affecting the basic life activities of cells and physiological functions, including cell movement and migration [62,63]

**Figure 3:**
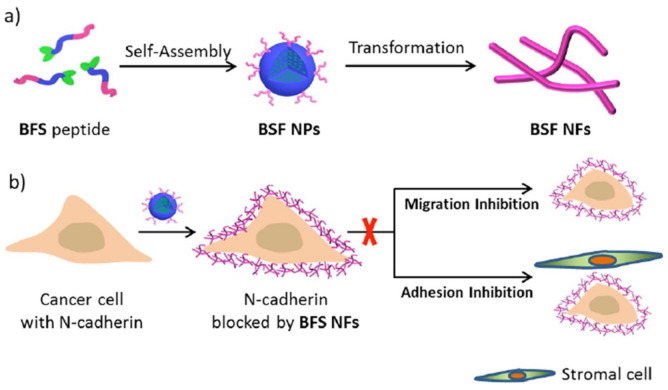
Schematic illustration of construction of peptide networks from transformable peptide nanoparticles by binding N-cadherin to inhibit migration and adhesion of cancer cells. (**a**) The peptide self-assembly into nanoparticles and transform into nanofibers upon binding N-cadherin. (**b**) The in situ construction of the peptide on N-cadherin cell surfaces to inhibit the adhesion and migration. Reproduced with permission from [67], Elsevier, 2020.

Tumors are the result of rapid proliferation of mutant cells in the body, where a large amount of oxygen and nutrient supplies, as well as various signals exchanged with extracellular tissues, are also required during this process. Therefore, cutting off the supply for a tumor could be an effective method for arresting tumor growth [64]. In recent years, some strategies, such as starvation treatment, achieved quite a curative response, which greatly boosted the morale of researchers [65]. Furthermore, self-assembly peptides have been proven to physically disrupt the cell membrane or inhibit cellular metabolism by facilitating self-assembly, polymerization, and biomineralization after entering the cells with an energy-independent pathway [66]. This would establish the groundwork for exploring a potent drug-free approach for treating cancer [61]. Inspired by the merits of self-assembled peptides, Fan et al. designed a transformable peptide, BP-KLVFF-SWTLYTPSGQSK (BFS), that can form peptide networks in situ precisely targeting to N-cadherin and high-efficiently blocking the N-cadherin as “a biomimetic antibody” to thereby, inhibit the migration of cancer cells in the end [67] (Figure 3). Meanwhile, this concept has also been applied in the suppression of tumor metastasis. Based on the self-assembled fibrils of KLVFF, a designer liposomal system was synthesized. It could spontaneously undergo self-assembly to form nanofibers with a net-like structure wrapping around tumor cells, and then bury the membrane protrusions and hinder the migration and invasion of tumor cells, especially the transmigration through the fenestrated endothelium. Hence, there will be a promising avenue to combat tumor metastasis by regulating the interactions between tumor cells and the tumor microenvironment (TME) [68]. Surprisingly, it was found that the optimum hydrophobic−hydrophilic balance played a crucial role in driving the self-assembly of amphiphilic peptides and even effected the target sites and selectivity for cancer cells. In addition to the strategy of “net bag restraint”, peptide-based NPs were successfully developed to initiate coagulation and form clots in blood vessels by mimicking the morphology transformation of platelets. After blocking the blood supply, tumor growth was inhibited effectively [69].

Furthermore, the cytoskeleton referring to the protein fiber network structure in eukaryotic cells not only plays a vital role in maintaining cell shape, bearing external forces and maintaining the order of internal cell structure, but also participates in many important life activities. Studies have reported that small-molecule self-assembly nanostructures could effectively inhibit the proliferation of cancer cells by selectively interacting with various proteins in the cell in a non-single way. Because of the biological significance of self-assembling peptides, our group fabricated the glutathione (GSH)-responsive PEG-Pep (FFKY) nanoparticles, which disintegrated in high GSH, and D-peptide FFKY as a self-assembling building block or hydrophobic section that could form nanofibers. The self-assembling nanofibers might prevent the assembly of actin in the cytoplasm and even damage the existing actin filaments in cells to, consequently, achieve the synergistic effect with doxorubicin [70,71]. Meanwhile, the mechanism of synergistic effects destroying the cell membrane system might be related to the formation mechanism of NLPR3 inflammasomes resulting from the disintegration of the Golgi’s reverse network structure in activation signal transmission and, thereby, initiate a new cell death pathway, pyroptosis [72]. The research may have demonstrated that a “drug-free approach” based on the self-assembled peptides is a promising therapeutic strategy for tumors.

### 3.3. Immune Adjuvants

The traditional treatment methods, such as chemotherapy or radiotherapy, are passive treatments relative to the entire tumor environment that aim to suppress tumor cell growth and break its “hard shell” [73]. However, this approach cannot effectively solve the treatment bottleneck of malignant melanoma metastasis and recurrence and even lead to the multidrug resistance that makes the tumor more difficult to eradicate. Nevertheless, the occurrence of tumor resistance is the result of suppressed immune system in the tumor site, therefore, alleviating immunosuppression would improve the body’s own anti-tumor activity [74]. Therefore, cancer immunotherapy, including immune checkpoint blockades, adoptive cell transfers, and vaccines, has attracted enormous preclinical and clinical studies over the past decade [75,76,77,78,79,80]. However, adjuvants such as CpG and monophosphoryl lipid A (MPLA), as well as alum utilized extensively to promote immune responses to vaccines, do not always generate high levels of Tfh cell responses and strong inflammatory responses. It would be difficult to balance with immunogenicity and even counterproductive with respect to vaccine efficacy, which limited its application in tumor therapy [81,82,83,84]. Fortunately, self-assembled peptides with superior performance have shown their potential in adjuvant immunotherapy, which provides evidence for the exploration of new immune adjuvants.

**Figure 4:**
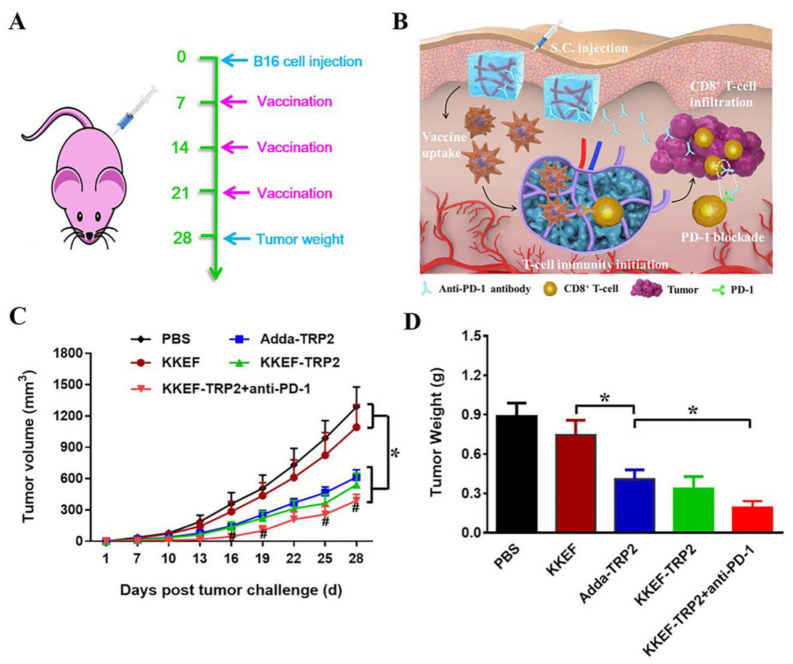
KKEF-TRP2 vaccine increases the therapeutic antitumor effect in melanoma model. (**A**) The protocol of tumor cell inoculation and vaccination. (**B**) Schematic of antitumor T-cell response induced by KKEF-TRP2 hydrogel through efficient peptide delivery and combination immunotherapy with anti-PD-1. (**C**) The tumor volume in the time range of 28 days after cell inoculation. (**D**) The mass weight of excised tumors at day 28. Values represent mean ± SDs (*n* = 6) from at least three independent experiments. * *p* < 0.05, between indicated groups; ^#^ *p* < 0.05, between KKEF-TRP2 and KKEF-TRP2+anti-PD-1 groups. Reproduced with permission from [85], John Wiley and Sons, 2019.

Recently, a proton-driven nanotransformer-based vaccine (NTV) comprised of a self-assembled polypeptide with the loaded antigenic peptide (AP) was reported, where the vaccine particles could transform into nanosheets in acidic media, causing endosomal membrane disruption so as to boost tumor immunity via activation of NLRP3-inflammasome pathways and enhance antigen processing in DCs [81]. Meanwhile, Wang et al. designed a set of GSH-responsive self-assembled peptide-based supramolecular hydrogels co-assembled vaccines (Fbp-GDFDFDYD (E, S, or K)-*ss*-ERGD). It was found that the peptide-based hydrogels effectively boosted antibody production and tumor curative effects, acting as novel vaccine adjuvants. Moreover, tuning the surface properties of self-assembling peptides could ultimately acquire different immune effects [82]. Except for the β-amyloid derived peptides, RADA16 peptide or Q11 (QQKFQFQFEQQ) or Coil29 were used to assemble the hydrogel scaffold for delivering exogenous DCs, antigens, and anti-PD-1 antibody in a minimally invasive manner [83]. Furthermore, the self-assembling peptide hydrogel was proven to prolong the cell duration time period of vaccines at the injection site and maintain their biological function, including antigen uptake, activation, and maturation, so as to increase the drainage to lymph nodes and stimulate a strong antigen-specific cellular response. More importantly, these nanofibers delivering vaccines (eg, OVA etc.) were able to elicit antibody responses with higher titers and avidities compared with conventional adjuvants alum or sigma adjuvant system (SAS). Additionally, relative to β-sheet fibrillar system, the α-helical nanofiber system was more inclined to raise stronger CD4+ T cell or B cell responses, as well as follicular helper T cell (Tfh) responses. These findings explore new possibilities for therapeutic peptide delivery and provide varied new strategies for cancer immunotherapy [84,85,86] (Figure 4).

### 3.4. Imaging/PDT/PTT

Cancer morbidity and mortality rates remain high worldwide [87]. So far, diagnostic tests, including physical examination, biopsy, imaging examination, and endoscopy, have been widely used clinically as powerful tools for sensor analysis and optical imaging to locate tumor cells. Moreover, the potential bioimaging fluorescent probes should be equipped with the characteristics of non-invasiveness, high sensitivity, real-time detection and simplicity, which could be preferred to distinguish tumors from normal tissues and directly image at the molecular level, as well as effectively gain insights into complex biological structures and physiology. However, achieving high bio-imaging sensitivity with specificity for the tumor, characterized by tissue barriers and heterogeneity, is still an arduous task [88,89,90,91]. Because of the inherent variable physical and chemical properties that benefit from their special polarity, charge, and hydrophobicity of the side chains of amino acids, self-assembled polypeptides have attracted intensive research activities in the bio-imaging field. Under certain conditions, they could carry photosensitizers or heat sensitizers and even interact with them by the non-covalent bonds so as to optimize their image properties [92]. For instance, a peptide-based near-infrared probe was constructed that was responsive to fibroblast activation protein-α (FAP-α) and specifically formed nanofibers on the surface of cancer-associated fibroblasts (CAFs) in situ. The assembly/aggregation-induced retention (AIR) effect led to enhanced accumulation of the probe around the tumor and amplified imaging signal compared to that of a control probe that does not aggregate. Based on the enhanced tumor imaging capability, this probe can visualize small tumors around 2 mm in diameter, which will provide strong guidance for clinical surgical treatment of tumors [93,94,95]. Furthermore, trypsin-responsive near-infrared fluorescent (NIRF) and magnetic resonance (MR) dual-imaging composite nanoparticle/polypeptide coacervate nanoprobes with tunable sizes have also been constructed herein via electrostatic interaction-induced self-assembly to efficiently map malignant tumors with overexpressed trypsin and control the delivery of targeted payloads. At the same time, the spontaneous functional self-assembly of peptides has been employed to trace the intracellular behavior of nanoformulations and validate the synergistic chemotherapy mechanism of self-assembled fibers [96].

Phototherapy can be mainly divided into photodynamic therapy (PDT) and photothermal therapy (PTT) according to the treatment mechanism, whether it relies on the excitation of photosensitive molecules through light to generate reactive oxygen species (ROS) or local hyperthermia to kill tumor cells [97,98,99]. Over the past decade, PDT with interesting optical properties have exhibited a lot of encouraging results in the in vitro and in vivo studies [100,101]. At present, a large number of studies have confirmed that the use of self-assembling peptides or protein nanomaterials as drug delivery systems can effectively improve the solubility and stability of hydrophobic phototherapeutics and reduce their non-specific phototoxicity. Meanwhile, the enhanced tumor accumulation through the EPR effect would effectively improve its therapeutic effect of phototherapy. A simple dipeptide- or amphiphilic amino acid-tuned self-assembly of photosensitizers (FF-PSs) was exploited; the assembled nanodrugs exhibited greatly improved PS loading efficiency based on the stoichiometry between PSs and amphiphilic dipeptides or amino acids, as well as preferable cellular uptake and biodistribution [102,103]. These features resulted in greatly enhanced PDT efficacy in vitro and in vivo, leading to almost complete tumor eradication in mice receiving a single drug dose and a single exposure to light. Therefore, these developed strategies, based on simple peptide-regulated self-assembly nanoagents, will provide a conceptually novel and promising platform toward PDT/PTT therapy of tumor.

### 3.5. Regenerative Medicine

Tissue engineering serves as a key approach for efficient reconstructive and regenerative medicine. In recent years, natural polysaccharide hydrogels or biological scaffolds equipped with the special intrinsic biocompatibility, permeability, and bio-restorability have become the ideal candidate materials for tissue engineering and have achieved milestones [104,105,106]. Among them, the self-assembled polypeptides have been widely explored, which could form diverse peptide-based architectures, including nanofibrils, nanotubes, nanosheets, and hydrogels, mimicking the extracellular matrix (ECM) and serving efficiently as 3D scaffolds [107,108]. Furthermore, as a benefit from its simple synthesis, possible in situ organization, and feasible chemical modifications, self-assembled peptides acting as the building blocks to load drugs and supporting scaffold have received in-depth study.

#### 3.5.1. Cell Culture

It was found that modifying the structure and combination of two or more peptide hydrogelators could change the intrinsic mechanical force and properties of self-assembled peptides [109,110]. Diaferia et al. reported the synthesis, formulation, and multi-scale characterization of peptide-based mixed hydrogels formed by the low molecular weight Fmoc-FF (Na-fluorenylmethyloxycarbonyl diphenylalanine) hydrogelator and the PEG8-(FY)_3_ hexapeptide, containing three repetitions of the Phe-Tyr motif and a PEG moiety at its N-terminus. Rheology analysis confirmed the improved mechanical features of the multicomponent gels prepared at two different ratios (2/1 or 1/1, *v/v*). Meanwhile, Michal Halperin-Sternfeld’s group demonstrated that the co-assembly of Fmoc-F_5_-Phe and Fmoc-FF in the 1:1 hybrid formulation exhibited remarkable mechanical properties with a storage modulus and an order of magnitude higher than hydrogels formed by each of the individual building blocks. The adequate rigidity for both cell attachment and mechanical support of these multicomponent hydrogels suggested a potential employment in cell proliferation as exogenous scaffold materials [111,112].

#### 3.5.2. Bone Tissue Regeneration

A part of peptide-based hydrogels as promising candidates for biomimetic scaffolds were limited due to their low mechanical properties in bone tissue engineering. Therefore, promoting the rigidity of peptide-based scaffolds would be the dominating research direction. Due to its excellent mechanical property, Fmoc-FF/Fmoc-R combination with the bone mineral hydroxyapatite, which mediated with high affinity to hydroxyapatite (HAP), could serve as functional biomaterials for improved bone regeneration. Moreover, Onak et al. designed self-assembling peptide scaffolds KLD (KLDLKLDLKLDL) through direct coupling to short bioactive motif O1 (EEGGC) and O2 (EEEEE) with bioactivity on osteogenic differentiation, which could enhance osteogenesis and biomineralization of injectable self-assembled hydrogels with controlled mechanical properties so as to be injected to bone defects. Compared with a pure KLD scaffold, these designed bioactive peptide scaffolds significantly promoted hMSCs proliferation depicted, the collagen type I (COL-1), and osteopontin (OP). Furthermore, osteocalcin (OCN) expression levels were also significantly increased with the addition of glutamic acid residues to KLD directed by biochemical analysis of alkaline phosphatase (ALP) activity and total calcium deposition. Therefore, these designed bioactive peptide scaffolds may be useful for promoting bone tissue regeneration [113,114].

#### 3.5.3. Neuron Repair and Regenerate

**Figure 5:**
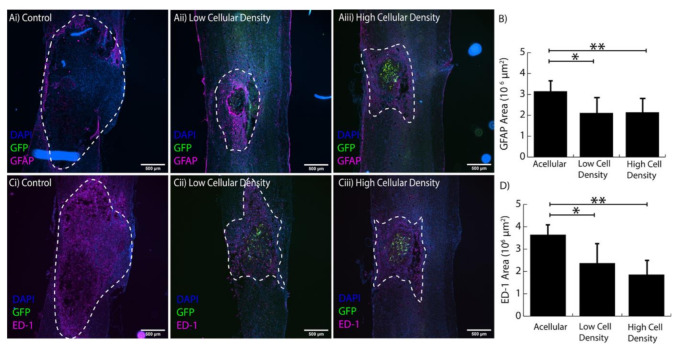
Quantification of injury area and inflammation following injection of peptide hydrogels. Reactive gliosis in response to peptide hydrogels in (**Ai**) acellular peptide, (**Aii**) low cell density, and (**Aiii**) high cell density. DAPI (blue), GFP (green), GFAP (magenta). (**B**) Injury area quantification. Data presented as mean ± s.d. * *p* < 0.05, ** *p* < 0.001 (**C**) Inflammatory response following scaffold transplantation, (**Ci**) acellular peptide, (**Cii**) low cell density, and (**Ciii**) high cell density. (**D**) Inflammation area quantification. Data presented as mean ± s.d. * *p* < 0.05, ** *p* < 0.001. Scale = 100-μm. (For interpretation of the references to color in this figure legend, the reader is referred to the web version of this article. Reproduced with permission from [86], Elsevier, 2020.

The disruption of the blood–spinal cord barrier (BSCB) following spinal cord injury contributes to inflammation and glial scarring that inhibits axon growth and diminishes the effectiveness of conduits transplanted to the injury site to promote this growth. Bioengineered scaffolds, which could bridge the damaged spinal cord so that patients can experience functional recovery, hold tremendous potential for stimulating axon growth in the aftermath of a spinal cord injury (SCI). Tran’s group used RADA-16I, the self-assembling peptide scaffold equipped with established permissiveness to axon growth and ability to support vascularization, to evaluate that the self-assembled scaffolds containing microvessels that exhibit BSCB integrity could reduce inflammation and scar formation at the injury site and increase the density of axons growing into the injury/transplant site (Figure 5). Furthermore, self-assembling peptide (FEFK)-based polymer functionalized with the thrombin-inhibiting peptides that can be released enzymatically by MMP3 could co-deliver the neural progenitor cells to achieve better engraftment of transplanted cells. Some investigators showed a panel of eight MDPs containing various motifs mimicking extracellular matrix components and growth factors would successfully self-assemble into injectable nanofibrous hydrogels. Various lysine based MDPs were found to enhance macrophage recruitment to the injury site and degrade efficiently over time so as to significantly accelerate functional recovery and remyelination in peripheral nerve injury. Meanwhile, a novel RADA16 self-assembling peptide scaffold integrating a neural-cell adhesion molecule-derived mimetic-peptide (SIDRVEPYSSTAQ) also promoted neuron proliferation and adherent ability and inhibited apoptosis, as well as stimulated neurite extension by reducing Tau protein phosphorylation through the calpain/GSK-3b signaling pathway. These proof-of-concept studies exhibited the potential prospects of these materials both in the peripheral and central nervous system [86,115,116,117].

#### 3.5.4. Wound Healing

Uncontrolled hemorrhaging remains a contributor of mortality in surgery, battle, and disaster emergencies, while chronic skin wound healing is also one of the major burdens of patients, seriously affecting the postoperative quality of life [118,119]. Self-assembled peptide hydrogels achieved complete hemostasis in a short time by forming nanofiber barriers and concentrating the tangible blood components when applied directly to wounds. Wei et al. synthesized a novel polypeptide material, RATEA16 (CH_3_CO–RAT–RAEARATARAEA–CONH_2_), that could form nanofibers induced by self-assembling behavior and showed a higher blood coagulation rate than CMS and achieved complete hemostasis in about 40 s. It has potential to serve as a reliable and promising hemostatic agent for rapid hemostasis. Furthermore, exhibiting good injectability and tunable mechanical properties, self-assembled peptides have been applied in chronic wound healing. The classical example is EAK16-II(AEAEAKAKAEAEAKAK), which self-assembled to form a hydrogel loading drug for performed sustained release. Recently, other types of SAPs, including crosslinked ultrashort peptides (LIVAGKC), multidomain peptides consisting of 16-amino acids of K2 (SL)_6_K2, and N-fluorenylmethyloxycarbonyl SAPs have been explored for wound healing. These self-assembled peptide nanomaterials, combined to target various aspects of the wound healing process, play a vital role in recovery [120,121,122]. Additionally, antimicrobial peptides or their simplified polypeptide analogues are showing great potential for combating multidrug-resistant bacteria and anti-infection therapy, which will greatly improve wound healing and speed up the recovery of the disease [123,124].

## 4. Expectation

Based on the advantages of the biological functions and structural characteristics of polypeptide molecules, the design, modification, and assembly of polypeptide monomers realize the modular biological functional integration of different functional polypeptide molecules, thereby constructing a polypeptide nanomedicine system with diverse biological functions. While taking the biocompatibility and biodegradability of peptides into account, we can comprehensively utilize the pathological and physiological characteristics of the disease microenvironment to accurately construct a peptide nanomedicine system so as to achieve controllable allosteric and targeted therapy in the tumor microenvironment. In summary, the peptide-based self-assembled system will open a new avenue for diverse biomedical applications.

## Figures and Tables

**Table 1:** Some representative self-assembled polypeptides applied in medicine discussed in this paper.

Polypeptides	Assembled Module	Responsive Type	Aggregations	Application
SLaM: K–(SL)6–K–G-WKNFQTI	K–(SL)_6_–K	shear-responsive	nanofibrils	immunomodulation
KFAK	H_2_N-KKFAFAFAKK-COOH	—	fibril to sheet	tissue engineering
PNIPAM-I3K	I3K	thermo-sensitive	nanofibrils	drug delivery
NapGDFDFpYSV	NapGDFDFp	enzyme-responsive	hydrogels	anti-tumor
Ade-FFF	Ade-FFF	—	hydrogels	drug delivery
FDPC	KIGLFRWR	pH-sensitive	nanofibers	drug delivery
P3	KLVFFAE	—	nanofibers	chemosensitization
NF/PDGF-BB	AcN-RARADADARARADADA-CNH_2_	—	nanofibers	drug delivery
BFS	KLVFF	—	nanofibers	anti-tumor
PEG-Pep-TPE	FFKY	GSH-responsive	nanofibers	chemotherapy synergy
Fbp-GDFDFDYD (E, S, or K)-*ss*-ERGD	Fbp-GDFDFDYD (E, S or K)-SH	GSH-responsive	nanofibers	immune adjuvants
O1/O2	KLDLKLDLKLDL	—	hydrogels	bone tissue regeneration
RADA16-FRM-MP	RADA16	—	nanofibers	neuron repair and regenerate
p(TEGMA-*co*-BM3)-FEFK	FEFK	MMP3-responsive	nanofibers	CNS regeneration
RADA-16I	RADA-16I	pH-sensitive	hydrogels	tissue regeneration
RATEA16	RATEA16	pH-responsive	nanofibers	wound healing
EAK16-II	AEAEAKAKAEAEAKAK	—	hydrogels	wound healing
K_2_MDP	K2 (SL)_6_K2	—	hydrogels	wound healing

## References

[1] Mazo A.R., Allison-Logan S., Karimi F., Chan J.A., Qiao G.G. (2020). Ring opening polymerization of α-amino acids: Advances in synthesis, architecture and applications of polypeptides and their hybrids. Chem. Soc. Rev..

[2] Colin B. (2018). Secondary structures of synthetic polypeptide polymers. Polym. Chem..

[3] Yang C., Liang G., Lin J., Wang L., Li Z. (2017). Toroid Formation through a Supramolecular “Cyclization Reaction” of Rodlike Micelles. Angew. Chem. Int. Ed. Engl..

[4] Abbas M., Zou Q., Li S., Yan X. (2017). Self-Assembled Peptide- and Protein-Based Nanomaterials for Antitumor Photodynamic and Photothermal Therapy. Adv. Mater..

[5] Wang W., Ma Z., Zhu S., Wan H., Yue J., Ma H., Ma R., Yang Q., Wang Z., Li Q. (2018). Molecular Cancer Imaging in the Second Near-Infrared Window Using a Renal-Excreted NIR-II Fluorophore-Peptide Probe. Adv. Mater..

[6] Mendes A.C., Baran E.T., Reis R.L., Azevedo H.S. (2013). Self-assembly in nature: Using the principles of nature to create complex nanobiomaterials. Wiley Interdiscip. Rev. Nanomed. Nanobiotechnol..

[7] Mitchison T., Kirschner M. (1984). Dynamic instability of microtubule growth. Nature.

[8] Mitchison T., Kirschner M. (1984). Microtubule assembly nucleated by isolated centrosomes. Nature.

[9] Brenner S., Jacob F., Meselson M. (1961). An Unstable Intermediate Carrying Information from Genes to Ribosomes for Protein Synthesis. Nature.

[10] Shi Y. (2002). Apoptosome: The cellular engine for the activation of caspase-9. Structure.

[11] Martinon F., Burns K., Tschopp J. (2002). The inflammasome: A molecular platform triggering activation of inflammatory caspases and processing of proIL-beta. Mol. Cell.

[12] Lashuel H.A., Hartley D., Petre B.M., Walz T., Lansbury P.T. (2002). Neurodegenerative disease: Amyloid pores from pathogenic mutations. Nature.

[13] Mann S. (2008). Life as a Nanoscale Phenomenon. Angew. Chem. Int. Ed..

[14] Grazon C., Salas-Ambrosio P., Ibarboure E., Buol A., Bonduelle C. (2019). Aqueous Ring-Opening Polymerization Induced Self-Assembly (ROPISA) of N-carboxyanhydrides. Angew. Chem. Int. Ed..

[15] Ruoslahti E. (2012). Peptides as Targeting Elements and Tissue Penetration Devices for Nanoparticles. Adv. Mater..

[16] Hofmann S., Bellmann-Sickert K., Beck-Sickinger A.G. (2019). Chemical modification of neuropeptide Y for human Y1 receptor targeting in health and disease. Biol. Chem..

[17] Zhang S. (2002). Emerging biological materials through molecular self-assembly. Biotechnol. Adv..

[18] Ghadiri M.R., Granja J.R., Milligan R.A., Mcree D.E., Khazanovich N. (1993). Self-assembling organic nanotubes based on a cyclic peptide architecture. Nature.

[19] Woolfson D.N., Bartlett G.J., Bruning M., Thomson A.R. (2012). New currency for old rope: From coiled-coil assemblies to alpha-helical barrels. Curr. Opin. Struct. Biol..

[20] Rhys G.G., Wood C.W., Lang E.J.M., Mulholland A.J., Brady R.L., Thomson A.R., Woolfson D.N. (2018). Maintaining and breaking symmetry in homomeric coiled-coil assemblies. Nat. Commun..

[21] Veggiani G., Sidhu S.S. (2019). Peptides meet ubiquitin: Simple interactions regulating complex cell signaling. Pept. Sci..

[22] Li L.L., Qiao S.L., Liu W.J., Ma Y., Wan D., Pan J., Wang H. (2017). Intracellular construction of topology-controlled polypeptide nanostructures with diverse biological functions. Nat. Commun..

[23] Kuang Y., Shi J., Li J., Yuan D., Alberti K.A., Xu Q., Xu B. (2014). Pericellular Hydrogel/Nanonets Inhibit Cancer Cells. Angew. Chem. Int. Ed..

[24] Kalmouni M., Al-Hosani S., Magzoub M. (2019). Cancer targeting peptides. Cell. Mol. Life Sci..

[25] Saket A., Vikas P., Vandana S. (2018). RGD Peptide as a Targeting Moiety for Theranostic Purpose: An Update Study. Int. J. Pept. Res. Ther..

[26] Tian X., Sun F., Zhou X.R., Luo S.Z., Chen L. (2015). Role of peptide self-assembly in antimicrobial peptides. J. Pept. Sci..

[27] Qi G.B., Gao Y.J., Wang L., Wang H. (2018). Self-Assembled Peptide-Based Nanomaterials for Biomedical Imaging and Therapy. Adv. Mater..

[28] Ganta S., Devalapally H., Shahiwala A., Amiji M. (2008). A review of stimuli-responsive nanocarriers for drug and gene delivery. J. Control. Release.

[29] Liu M., Du H., Zhang W., Zhai G. (2016). Internal stimuli-responsive nanocarriers for drug delivery: Design strategies and applications. Mater. Sci. Eng. C.

[30] Kim K.K., Siddiqui Z., Patel M., Sarkar B., Kumar V.A. (2020). A self-assembled peptide hydrogel for cytokine sequestration. J. Mater. Chem. B.

[31] Jin H., Zhang L., Song Y., Liu L. (2019). Modulation of structure and mechanical properties of self-assembled peptide nanofibrils and nanosheets. Mater. Lett..

[32] Calvelo M., Granja J.R., Fandino R.G. (2019). Competitive double-switched Self-Assembled Cyclic Peptide Nanotubes: A dual internal and external control. Phys. Chem. Chem. Phys..

[33] Cao M., Wang Y., Hu X., Gong H., Lu J.R. (2019). Reversible Thermoresponsive Peptide-PNIPAM Hydrogels for Controlled Drug Delivery. Biomacromolecules.

[34] Gao Y., Zhang C., Chang J., Yang C., Liu J., Fan S., Ren C. (2019). Enzyme-instructed self-assembly of a novel histone deacetylase inhibitor with enhanced selectivity and anticancer efficiency. Biomater. Sci..

[35] Wiwanitkit V., Sereemaspun A., Rojanathanes R. (2009). Effect of gold nanoparticles on spermatozoa: The first world report. Fertil. Steril..

[36] De Jong W.H., Borm P.J. (2008). Drug delivery and nanoparticles: Applications and hazards. Int. J. Nanomed..

[37] Vega-Villa K.R., Takemoto J.K., Yáñez J.A., Remsberg C.M., Forrest M.L., Davies N.M. (2008). Clinical toxicities of nanocarrier systems. Adv. Drug Deliv. Rev..

[38] Cai C., Lin J., Lu Y., Zhang Q., Wang L. (2016). Polypeptide self-assemblies: Nanostructures and bioapplications. Chem. Soc. Rev..

[39] Shi J., Xu B. (2015). Nanoscale Assemblies of Small Molecules Control the Fate of Cells. Nano Today.

[40] Zhang C., Lu J., Tian F., Li L., Hou Y., Wang Y., Sun L., Shi X., Lu H. (2019). Regulation of the cellular uptake of nanoparticles by the orientation of helical polypeptides. Nano Res..

[41] Baek K., Noblett A.D., Ren P., Suggs L.J. (2020). Self-assembled nucleo-tripeptide hydrogels provide local and sustained doxorubicin release. Biomater. Sci..

[42] Xu L., Wang Y., Zhu C., Ren S., Chen Z. (2020). Morphological transformation enhances Tumor Retention by Regulating the Self-assembly of Doxorubicin-peptide Conjugates. Theranostics.

[43] Wang H., Feng Z., Tan W., Xu B. (2019). Assemblies of d-Peptides for Targeting Cell Nucleolus. Bioconjug. Chem..

[44] Naito S., Sakamoto N., Kotoh S., Goto K., Kumazawa J. (1993). Expression of P-glycoprotein and multidrug resistance in renal cell carcinoma. Eur. Urol..

[45] Wang Z., An H.-W., Hou D., Wang M., Zeng X., Zheng R., Wang L., Wang K., Wang H., Xu W. (2019). Addressable Peptide Self-Assembly on the Cancer Cell Membrane for Sensitizing Chemotherapy of Renal Cell Carcinoma. Adv. Mater..

[46] Wang F., Su H., Xu D., Dai W., Cui H. (2020). Tumour sensitization via the extended intratumoural release of a STING agonist and camptothecin from a self-assembled hydrogel. Nat. Biomed. Eng..

[47] Liu X., Feng Z., Wang C., Su Q., Song H., Zhang C., Huang P., Liang X.J., Dong A., Kong D. (2020). Co-localized delivery of nanomedicine and nanovaccine augments the postoperative cancer immunotherapy by amplifying T-cell responses. Biomaterials.

[48] Skoulas D., Stuttgen V., Gaul R., Cryan S.A., Heise A. (2020). Amphiphilic Star Polypept(o)ides as Nanomeric Vectors in Mucosal Drug Delivery. Biomacromolecules.

[49] Schatz C., Louguet S., Meins J.L., Lecommandoux S. (2009). Polysaccharide-block-polypeptide Copolymer Vesicles: Towards Synthetic Viral Capsids. Angew. Chem. Int. Ed..

[50] Oliveira H.D., Thevenot J., Garanger E., Ibarboure E., Lecommandoux S. (2014). Nano-Encapsulation of Plitidepsin: In Vivo Pharmacokinetics, Biodistribution, and Efficacy in a Renal Xenograft Tumor Model. Pharm. Res..

[51] Salva R., Meins J., Sandre O., BrûLet A., Schmutz M., Guenoun P., Lecommandoux S. (2013). Polymersome shape transformation at the nanoscale. ACS Nano.

[52] Klok H.A., Langenwalter J.F., Lecommandoux S. (2000). Self-Assembly of Peptide-Based Diblock Oligomers. Macromolecules.

[53] Caillol S., Lecommandoux S., Mingotaud A.F., Schappacher M., Soum A., Bryson N., Meyrueix R. (2009). Synthesis and Self-Assembly Properties of Peptide-Polylactide Block Copolymers. Macromolecules.

[54] Bellomo E.G., Wyrsta M., Pakstis L., Pochan D.J., Deming T.J. (2004). Stimuli-responsive polypeptide vesicles by conformation-specific assembly. Nat. Mater..

[55] Cameron N., Deming T. (2015). Peptide-based materials for nanomedicine. Macromol. Biosci..

[56] Hsieh P.C., Davis M.E., Gannon J., MacGillivray C., Lee R.T. (2006). Controlled delivery of PDGF-BB for myocardial protection using injectable self-assembling peptide nanofibers. J. Clin. Investig..

[57] Mcgovern S.L., Caselli E., Grigorieff N., Shoichet B.K. (2002). A Common Mechanism Underlying Promiscuous Inhibitors from Virtual and High-Throughput Screening. J. Med. Chem..

[58] Dobson C.M. (2003). Protein Folding and Misfolding. Nature.

[59] Kayed R. (2003). Common Structure of Soluble Amyloid Oligomers Implies Common Mechanism of Pathogenesis. Science.

[60] Zorn J.A., Wolan D.W., Agard N.J., Wells J.A. (2012). Fibrils Colocalize Caspase-3 with Procaspase-3 to Foster Maturation. J. Biol. Chem..

[61] Kuang Y., Du X., Zhou J., Xu B. (2014). Supramolecular nanofibrils inhibit cancer progression in vitro and in vivo. Adv. Healthc. Mater..

[62] Kuang Y., Long M.J.C., Zhou J., Shi J., Gao Y., Xu C., Hedstrom L., Xu B. (2014). Prion-like Nanofibrils of Small Molecules (PriSM) Selectively Inhibit Cancer Cells by Impeding Cytoskeleton Dynamics. J. Biol. Chem..

[63] Gao Y., Kuang Y., Du X., Zhou J., Chandran P., Horkay F., Xu B. (2013). Imaging Self-Assembly Dependent Spatial Distribution of Small Molecules in a Cellular Environment. Langmuir.

[64] Mäe M., Myrberg H., Langel E.A. (2009). Design of a Tumor Homing Cell-Penetrating Peptide for Drug Delivery. Int. J. Pept. Res. Ther..

[65] Cao J., Qiao B., Luo Y., Cheng C., Yang A., Wang M., Yuan X., Fan K., Li M., Wang Z. (2020). A multimodal imaging-guided nanoreactor for cooperative combination of tumor starvation and multiple mechanism-enhanced mild temperature phototherapy. Biomater. Sci..

[66] Jeena M.T., Palanikumar L., Go E.M., Kim I., Kang M.G., Lee S., Park S., Choi H., Kim C., Jin S.M. (2017). Mitochondria localization induced self-assembly of peptide amphiphiles for cellular dysfunction. Nat. Commun..

[67] Fan J., Fan Y., Wei Z., Li Y., Wang H. (2020). Transformable peptide nanoparticles inhibit the migration of N-cadherin overexpressed cancer cells. Chin. Chem. Lett..

[68] Luo S., Feng J., Xiao L., Guo L., Deng L., Du Z., Xue Y., Song X., Sun X., Zhang Z. (2020). Targeting self-assembly peptide for inhibiting breast tumor progression and metastasis. Biomaterials.

[69] Yang P.P., Zhang K., He P.P., Fan Y., Wang H. (2020). A biomimetic platelet based on assembling peptides initiates artificial coagulation. Sci. Adv..

[70] Wang T.-T., Wei Q.-C., Zhang Z.-T., Lin M.-T., Chen J.-J., Zhou Y., Guo N.-N., Zhong X.C., Xu W.-H., Liu Z.-X. (2019). AIE/FRET-based versatile PEG-Pep-TPE/DOX nanoparticles for cancer therapy and real-time drug release monitoring. Biomater. Sci..

[71] Zhan J., Cai Y., He S., Wang L., Yang Z. (2017). Tandem Molecular Self-Assembly in Liver Cancer Cells. Angew. Chem..

[72] Chen J., Chen Z.J. (2018). PtdIns4P on dispersed trans-Golgi network mediates NLRP3 inflammasome activation. Nature.

[73] Ajithkumar T., Parkinson C., Fife K., Corrie P., Jefferies S. (2015). Evolving treatment options for melanoma brain metastases. Lancet Oncol..

[74] Couzin-Frankel J. (2013). Breakthrough of the year 2013. Cancer immunotherapy. Science.

[75] Mellman I., Coukos G., Dranoff G. (2011). Cancer Immunotherapy Comes of Age. Nature.

[76] Cai J., Wang H., Wang D., Li Y. (2019). Improving Cancer Vaccine Efficiency by Nanomedicine. Adv. Biosyst..

[77] Gotwals P., Cameron S., Cipolletta D., Cremasco V., Crystal A., Hewes B., Mueller B., Quaratino S., Sabatos-Peyton C., Petruzzelli L. (2017). Prospects for combining targeted and conventional cancer therapy with immunotherapy. Nat. Rev. Cancer Clin. Oncol..

[78] Sharma P., Allison J.P. (2015). Immune checkpoint targeting in cancer therapy: Toward combination strategies with curative potential. Cell.

[79] Mueller K.L. (2015). Realizing the Promise. Science.

[80] Bonner J. (2001). Cancer vaccines. Chem. Ind..

[81] Martins K.A.O., Cooper C.L., Stronsky S.M., Norris S.L.W., Kwilas S.A., Steffens J.T., Benko J.G., Tongeren S.A.V., Bavari S. (2016). Adjuvant-enhanced CD4 T Cell Responses are Critical to Durable Vaccine Immunity. Ebiomedicine.

[82] Annalisa C., Elena P., Fabio F., Gabiria P., Peter A., Gianni P., Donata M. (2016). Modulation of Primary Immune Response by Different Vaccine Adjuvants. Front. Immunol..

[83] Reed S.G., Tomai M., Gale M.J. (2020). New horizons in adjuvants for vaccine development. Curr. Opin. Immunol..

[84] Munir A., Javier T., Roesler A.S., Pietro M., Billur A., Theall B.P., Haewon S., Mirna P., Margery S., Juraj K. (2018). Second signals rescue B cells from activation-induced mitochondrial dysfunction and death. Nat. Immunol..

[85] Yang P., Song H., Feng Z., Wang C., Huang P., Zhang C., Kong D., Wang W. (2019). Synthetic, Supramolecular, and Self-Adjuvanting CD8+ T-Cell Epitope Vaccine Increases the Therapeutic Antitumor Immunity. Adv. Ther..

[86] Tran K.A., Partyk P.P., Jin Y., Bouyer J., Galie P.A. (2020). Vascularization of self-assembled peptide scaffolds for spinal cord injury repair. Acta Biomater..

[87] Siegel R.L., Miller K.D., Jemal A. (2018). Cancer statistics, 2018. CA A Cancer J. Clin..

[88] Pyonteck S.M., Akkari L., Schuhmacher A.J., Bowman R.L., Sevenich L., Quail D.F., Olson O.C., Quick M.L., Huse J.T., Teijeiro V. (2013). CSF-1R inhibition alters macrophage polarization and blocks glioma progression. Nat. Med..

[89] Zhang J., Liu J. (2013). Tumor stroma as targets for cancer therapy. Pharmacol. Ther..

[90] Marusyk A., Almendro V., Polyak K. (2012). INtra-tumour heterogeneity: A looking glass for cancer?. Nat. Rev. Cancer.

[91] Maeda H., Nakamura H., Fang J. (2013). The EPR effect for macromolecular drug delivery to solid tumors: Improvement of tumor uptake, lowering of systemic toxicity, and distinct tumor imaging in vivo. Adv. Drug Deliv. Rev..

[92] Zou Q., Chang R., Xing R., Yuan C., Yan X. (2020). Injectable self-assembled bola-dipeptide hydrogels for sustained photodynamic prodrug delivery and enhanced tumor therapy. J. Control. Release.

[93] Zhang D., Qi G.-B., Zhao Y.-X., Qiao S.-L., Yang C. (2015). In Situ Formation of Nanofibers from Purpurin18-Peptide Conjugates and the Assembly Induced Retention Effect in Tumor Sites. Adv. Mater..

[94] Ye D., Shuhendler A.J., Cui L., Tong L., Tee S.S., Tikhomirov G., Felsher D.W., Rao J. (2014). Bioorthogonal cyclization-mediated in situ self-assembly of small-molecule probes for imaging caspase activity in vivo. Nat. Chem..

[95] Zhao X.-X., Li L.-L., Zhao Y., An H.-W., Cai Q., Lang J.-Y., Han X.-X., Peng B., Fei Y., Liu H. (2019). In Situ Self-Assembled Nanofibers Precisely Target Cancer-Associated Fibroblasts for Improved Tumor Imaging. Angew. Chem. Int. Ed..

[96] Guo H., Song S., Dai T., Sun K., Dou H. (2020). Near-Infrared Fluorescent and Magnetic Resonance Dual-Imaging Nano-Coacervates for Trypsin Mapping and Targeted Payload Delivery of Malignant Tumors. ACS Appl. Mater. Interfaces.

[97] Li S., Zhang W.J.C.M., Xue H., Xing R., Yan X. (2020). Tumor microenvironment-oriented adaptive nanodrugs based on peptide self-assembly. Chem. Sci..

[98] Chu C., Lin H., Liu H., Wang X., Wang J., Zhang P., Gao H., Huang C., Zeng Y., Tan Y. (2017). Tumor Microenvironment-Triggered Supramolecular System as an In Situ Nanotheranostic Generator for Cancer Phototherapy. Adv. Mater..

[99] Liu J., Shi J., Nie W., Wang S., Liu G., Cai K. (2020). Recent Progress in the Development of Multifunctional Nanoplatform for Precise Tumor Phototherapy. Adv. Healthc. Mater..

[100] Sun W., Zhao X., Fan J., Du J., Peng X. (2019). Photodynamic Therapy: Boron Dipyrromethene Nano-Photosensitizers for Anticancer Phototherapies. Small.

[101] Cheng L., Wang C., Feng L., Yang K., Liu Z. (2014). Functional Nanomaterials for Phototherapies of Cancer. Chem. Rev..

[102] Da Silva E.R., Faria de Freitas Z.M., Brito Gitirana L.D., Ricci-Júnior E. (2010). Improving the topical delivery of zinc phthalocyanine using oleic acid as a penetration enhancer:in vitropermeation and retention. Drug Dev. Ind. Pharm..

[103] Liu K., Xing R., Zou Q., Ma G., Möhwald H., Yan X. (2016). Simple Peptide-Tuned Self-Assembly of Photosensitizers towards Anticancer Photodynamic Therapy. Angew. Chem..

[104] Adler-Abramovich L., Gazit E. (2014). The physical properties of supramolecular peptide assemblies: From building block association to technological applications. Chem. Soc. Rev..

[105] Lee J., Choe I.R., Kim N.K., Kim W.J., Jang H.S., Lee Y.S., Nam K.T. (2016). Water-Floating Giant Nanosheets from Helical Peptide Pentamers. ACS Nano.

[106] Jang H.S., Lee J.H., Park Y.S., Kim Y.O., Park J., Yang T.Y., Jin K., Lee J., Park S., You J.M. (2014). Tyrosine-mediated two-dimensional peptide assembly and its role as a bio-inspired catalytic scaffold. Nat. Commun..

[107] Zehnder T., Freund T., Demir M., Detsch R., Boccaccini A.R. (2016). Fabrication of Cell-Loaded Two-Phase 3D Constructs for Tissue Engineering. Materials.

[108] Brown J.H., Das P., Divito M.D., Ivancic D., Poh Tan L., Wertheim J.A. (2018). Nanofibrous PLGA electrospun scaffolds modified with type I collagen influence hepatocyte function and support viability in vitro. Acta Biomater..

[109] Diaferia C., Ghosh M., Sibillano T., Gallo E., Stornaiuolo M., Giannini C., Morelli G., Adler-Abramovich L., Accardo A. (2019). Fmoc-FF and hexapeptide-based multicomponent hydrogels as scaffold materials. Soft Matter.

[110] Halperin-Sternfeld M., Ghosh M., Sevostianov R., Grigoriants I., Adler-Abramovich L. (2017). Molecular co-assembly as a strategy for synergistic improvement of the mechanical properties of hydrogels. Chem. Commun..

[111] Song S., Wang J., Cheng Z., Yang Z., Shi L., Yu Z. (2020). Directional molecular sliding movement in peptide hydrogels accelerates cell proliferation. Chem. Sci..

[112] Restu W.K., Yamamoto S., Nishida Y., Ienaga H., Maruyama T. (2020). Hydrogel formation by short D-peptide for cell-culture scaffolds. Mater. Sci. Eng. C.

[113] Ghosh M., Halperin-Sternfeld M., Grigoriants I., Lee J., Nam K.T., Adler-Abramovich L. (2017). Arginine-Presenting Peptide Hydrogels Decorated with Hydroxyapatite as Biomimetic Scaffolds for Bone Regeneration. Biomacromolecules.

[114] Onak G., Gkmen O., Yaral Z.B., Karaman O. (2020). Enhanced Osteogenesis of Human Mesenchymal Stem Cells by Self-Assembled Peptide Hydrogel Functionalized with Glutamic Acid Templated Peptides. J. Tissue Eng. Regen. Med..

[115] Koutsopoulos S., Zhang S. (2013). Long-term three-dimensional neural tissue cultures in functionalized self-assembling peptide hydrogels, Matrigel and Collagen I. Acta Biomater..

[116] Lee J., Zhao T., Peeler D.J., Lee D.C., Pun S.H. (2020). Formulation of thrombin-inhibiting hydrogels via self-assembly of ionic peptides with peptide-modified polymers. Soft Matter.

[117] Silva L., Cristobal C.D., Lai C.S.E., Aranda L., Lee H.K., Hartgerink J.D. (2020). Self-assembling multidomain peptide hydrogels accelerate peripheral nerve regeneration after crush injury. Biomater. Sci..

[118] Behrens A.M., Sikorski M.J., Kofinas P. (2014). Hemostatic strategies for traumatic and surgical bleeding. J. Biomed. Mater. Res. Part A.

[119] Kang H.J., Chen N., Dash B.C., Hsia H.C., Berthiaume F. (2020). Self-Assembled Nanomaterials for Chronic Skin Wound Healing. Adv. Wound Care.

[120] Wei S., Chen F., Geng Z., Cui R., Liu C. (2020). Self-assembling RATEA16 peptide nanofiber designed for rapid hemostasis. J. Mater. Chem. B.

[121] Zhao C., Zhu L., Wu Z., Yang R., Liang L. (2019). Resveratrol-loaded peptide-hydrogels inhibit scar formation in wound healing through suppressing inflammation. Regen. Biomater..

[122] Xu X.D., Liang L., Cheng H., Wang X.H., Zhang X.Z. (2012). Construction of therapeutic glycopeptide hydrogel as a new substitute for antiproliferative drugs to inhibit postoperative scarring formation. J. Mater. Chem..

[123] Shu J.L., O’Brien-Simpson N.M., Pantarat N., Sulistio A., Qiao G.G. (2016). Combating multidrug-resistant Gram-negative bacteria with structurally nanoengineered antimicrobial peptide polymers. Nat. Microbiol..

[124] Salas-Ambrosio P., Tronnet A., Verhaeghe P., Bonduelle C. (2021). Synthetic Polypeptide Polymers as Simplified Analogues of Antimicrobial Peptides. Biomacromolecules.

